# Identifying factors associated with thriving after interpersonal trauma in a Canadian sample of adults

**DOI:** 10.1177/13591053261433238

**Published:** 2026-03-30

**Authors:** Martine Hébert, Victoria Banyard, Xiafei Wang, Ophélie Dassylva, Stéphanie El Asmar, Sherry Hamby

**Affiliations:** 1 Université du Québec à Montréal, QC, Canada; 2 Rutgers University, New Brunswick, NJ, USA; 3 University of Kentucky, Lexington, KT, USA; 4 Université de Montréal, QC, Canada; 5 University of the South & Life Paths Research Center, Sewanee, TN, USA

**Keywords:** strengths, interpersonal trauma, subjective well-being, health-related quality of life, post-traumatic growth

## Abstract

Resilience following interpersonal trauma is increasingly conceptualized as a multidimensional process grounded in psychosocial strengths, yet integrative frameworks bridging distinct strength-based models remain limited. This study integrated the Values in Action Inventory of Strengths with the Resilience Portfolio Model to examine resilience after interpersonal trauma. A sample of 672 English-speaking Canadian adults completed measures of victimization, psychosocial strengths, and indicators of thriving. Most participants (93.2%) reported at least one form of interpersonal victimization, and half experienced institutional betrayal. Using chi-square and multivariate logistic regression analyses, results showed a gradient effect; a higher number of strengths being associated with greater odds of thriving. Hope, sense of purpose, endurance, prudence, perspective, and social support predicted well-being, while zest and humility predicted health-related quality of life. Post-traumatic growth was associated with purpose, hope, endurance, social support, and love. Findings underscore the differentiated roles of strengths in adaptation following adversity and provide guidance for resilience-informed interventions.

## Introduction

Interpersonal trauma is a major global health concern associated with a plethora of adverse consequences. Despite the numerous harmful effects on victims’ mental and physical health, responses to childhood victimization and interpersonal trauma vary significantly into adulthood. While many individuals experience severe consequences, others benefit from supportive contexts and resources that foster resilience in the face of adversity. This variability has driven extensive research into resilience and calls for studying strengths globally, across varied communities and contexts. This study aimed to examine adversities and strengths in a Canadian sample using the Values in Action (VIA) Inventory of Strengths-Positive Assessment (Peterson and Seligman, 2004) and the Resilience Portfolio Model (RPM; Grych et al., 2015). It sought to explore the strengths associated with thriving, including subjective well-being, health-related quality of life, and post-traumatic growth.

### Interpersonal trauma

Interpersonal trauma encompasses many harmful experiences including sexual abuse, physical and emotional violence, neglect, exposure to partner violence, and bullying that predominantly occur during childhood or adolescence within relational contexts (Finkelhor et al., 2007). Additionally, interpersonal trauma extends to community violence and is increasingly recognized to also extend to institutional betrayals. Institutional betrayal refers to the failure of institutions, such as universities or workplaces, to take appropriate measures in preventing or adequately responding to instances of interpersonal trauma (Smith and Freyd, 2014). This specific form of victimization has garnered increasing attention in the scientific community due to its high prevalence, particularly in military contexts and post-secondary academic settings (e.g. universities and colleges), and its association with various adverse effects, including burnout, post-traumatic stress symptoms, depression, and suicidality (Christl et al., 2024). Although institutional betrayal may occur later in development, exposure within educational and occupational contexts can compound the effects of earlier interpersonal trauma experienced during childhood and adolescence by undermining confidence in systems intended to protect individuals, thereby exerting long-term effects on well-being and growth.

Interpersonal trauma is highly prevalent. In Canada, a nationally representative study found that 32% of adults reported experiencing physical or sexual abuse or exposure to violence during childhood (Afifi et al., 2014). Such adversities rarely occur in isolation. Indeed, research indicates that children who experience one form of victimization are at an increased risk of experiencing multiple types of victimization (Finkelhor et al., 2015). Consequently, studying victimization as a singular phenomenon fails to capture its often complex and cumulative nature.

One of the most consistent findings in trauma research is the diversity of adaptation profiles, with some individuals demonstrating positive adjustment despite adversity. Research has shown that this adaptation to trauma is not a rare or exceptional occurrence, but may rather reflect a normative phenomenon (Masten, 2001). This variability underscores the importance of identifying strengths and resources that support thriving following childhood trauma, which is the central aim of the present study.

### Resilience after experiences of interpersonal trauma

The concept of resilience presents both theoretical and methodological challenges (Bonanno, 2012; Grych et al., 2015). Early research often viewed resilience as a stable trait, suggesting the existence of a “resilient type” (e.g. (Connor and Davidson, 2003). However, personality accounts for only a small portion of behavioral variability (Bonanno, 2012). Resilience is better understood as a dynamic process, shaped by individual differences in responses to early-life adversity and influenced by the risk and protective factors available at a given time (Rutter, 2012). To date, less is known about potential protective factors that could contribute to such resilience; more studies having focused on risk factors (Banyard et al., 2017). Existing work nevertheless highlights social support, emotion regulation, and meaning-making as key protective factors, although these are typically examined in isolation rather than within an integrative framework.

Moreover, most studies have defined resilience through the absence of psychopathology as a marker of positive adaptation, rather than the presence of well-being. This narrow focus adds little to the field, as it merely reframes non-pathology without offering deeper insights. Importantly, the absence of psychological disorders in the face of adversity does not necessarily reflect positive adjustment as people strive not just to avoid distress but to experience joy, hope, and fulfillment (Hamby et al., 2018). Despite this, relatively few studies have explored domains of functioning such as subjective well-being, posttraumatic growth, and health-related quality of life. These variables integrate psychopathology and well-being indicators, including positive affect and life satisfaction (Grych et al., 2015). While resilience reflects the ability to maintain psychological stability after adversity, posttraumatic growth involves positive transformative changes resulting from the process of overcoming significant life challenges (Tedeschi and Calhoun, 2004).

### Positive psychology and resilience

In the context of interpersonal trauma, both adversity exposure and protective strengths can be understood as operating across multiple ecological levels, from individual psychological processes to broader relational and social contexts. In recent years, the field of positive psychology has made significant contributions to the study of resilience by shifting focus from a deficit-based perspective to one that emphasizes human strengths and flourishing. This approach provides a conceptual framework that centers on the study of human strengths and virtues, aiming to understand and promote positive developmental outcomes (Seligman and Csikszentmihalyi, 2014). Positive psychology plays a pivotal role in fostering resilience by underlining strength-based approaches to coping and thriving in the face of adversity. Resilience is not merely about bouncing back from hardship; it also involves growth, adaptation, and the development of psychological resources (Masten and Cicchetti, 2016). In this regard, it is closely connected to constructs within positive psychology, such as optimism, emotion regulation, and social support, which help individuals build the resources needed to navigate life’s challenges effectively (Wu et al., 2013).

Within positive psychology, resilience is conceptualized not only as recovery from adversity but as the capacity to sustain or regain well-being through the activation of enduring psychological resources. Central to this perspective are character strengths, which represent positive, morally valued traits that support adaptive functioning across contexts (Peterson and Seligman, 2004). In the VIA framework, virtues are higher-order constructs reflecting core moral qualities (e.g. humanity, transcendence), while character strengths represent the specific, observable ways in which these virtues are expressed in everyday thoughts, emotions, and behaviors. From this perspective, virtues are enacted *through* character strengths rather than operating as distinct or separate processes. These strengths constitute key mechanisms through which positive psychology links individual characteristics to well-being and resilience. Empirical work has consistently shown that strengths such as hope, gratitude, and optimism are associated with higher subjective well-being and more adaptive responses to adversity, underscoring their role as resilience-promoting resources within a positive psychology framework (Sin and Lyubomirsky, 2009).

In trauma research, there is a growing interest in exploring factors associated with thriving. The RPM (Grych et al., 2015; Hamby et al., 2018) integrates advancements in resilience research and draws from positive psychology and posttraumatic growth research to better understand how individuals exposed to interpersonal trauma adapt. Grounded in a socio-ecological framework (Bronfenbrenner, 1977), the model incorporates protective factors, strengths, and resources at individual, familial, social, and community levels. This model frames resilience as a process, where well-being serves as a primary indicator rather than merely the absence of psychopathology. Aligned with a process-oriented perspective, this framework systematically integrates multiple protective factors that contribute to resilience, identifying potential targets for prevention and intervention strategies.

The model evaluates four functional categories of strengths (Banyard et al., 2025; Hamby et al., 2018). Regulatory strengths encompass abilities involved in managing emotions, thoughts, and behaviors, including psychological endurance and emotion regulation. Interpersonal strengths involve relational skills and support from broader social networks. Meaning-making strengths reflect an individual’s capacity for personal growth, such as finding meaning in adversity. Finally, environmental resources, such as time spent in nature, have been incorporated into the model (Banyard et al., 2025; Hamby et al., 2022). Rather than relying on a single factor, an individual’s adaptation to adversity is shaped by the overall constellation of assets and resources within their “portfolio” (Grych et al., 2015). This comprehensive perspective is captured by the concept of “poly-strengths,” which parallels the notion of “poly-victimization” used in research on the cumulative impact of violence (Finkelhor et al., 2007). This framework is particularly relevant to understanding adaptation to interpersonal trauma, as exposure to multiple forms of victimization can erode various protective factors that might otherwise support resilience (Grych et al., 2015).

Resilience in Canadian contexts has often been framed as a socially embedded and collective process rather than a purely individual trait. Sociological and policy traditions emphasize social solidarity, shared responsibility, and institutional support (e.g. universal health care, public education), shaping narratives of adaptation that extend beyond personal coping. Anthropological and Indigenous scholarship further conceptualizes resilience as relational continuity and cultural survivance, grounded in collective identity and historical context rather than individual recovery alone (Kirmayer et al., 2011). Together, these perspectives suggest that Canada provides a particularly relevant context for examining portfolio-based models of resilience that integrate personal strengths with interpersonal and structural resources.

While the RPM has been studied in various populations (Gonzalez-Mendez et al., 2021; Williams-Butler et al., 2024), its applicability to Canadian adults remains underexplored, despite distinct sociocultural, economic, and policy contexts that shape resilience processes. The Canadian health care system, based on universal access to medical services, directly influences health-related quality of life (Thomas and Sands, 2023), yet persistent inequalities, particularly among marginalized groups and those living in rural or northern regions, may differentially constrain access to resources that support adaptation. Although Canadian mental health policies are evolving, gaps remain in timely access to psychological services. A recent study among Canadian undergraduate students (Fang et al., 2025) provides preliminary support for the RPM, showing that both internal assets (e.g. emotion regulation) and external resources (e.g. social network supportiveness) predicted better mental health outcomes; however, academic contexts limit generalizability to broader adult populations. Extending the RPM to a broader Canadian adult sample may therefore help identify culturally relevant protective factors and strengthen the model’s broader applicability. Additionally, cross-cultural validation through applications to multiple global settings would further strengthen the model’s empirical foundation. Integrating the VIA-IS framework with the RPM may also offer a comprehensive approach to resilience-building interventions, particularly following interpersonal trauma. This integration could inform regionally applicable strategies for fostering psychological resources and enhancing positive adaptation.

Using the conceptual frameworks of the VIA-IS and the RPM, this study aimed to examine the unique contributions of psychosocial strengths following adversities to three key indicators: subjective well-being, post-traumatic growth, and health-related quality of life in a sample of adults from the general Canadian population. Integrating the VIA framework with the RPM allows for a more comprehensive understanding of resilience following interpersonal trauma. While the VIA framework identifies core individual character strengths associated with well-being, it does not explicitly situate these strengths within an adversity-exposed, socio-ecological resilience process. In contrast, the RPM emphasizes resilience as a dynamic outcome shaped by cumulative strengths and resources operating alongside adversity. Combining these frameworks enables examination of whether character strengths retain explanatory power when embedded in a trauma-informed, portfolio-based model of resilience, rather than functioning solely as general predictors of well-being.

We hypothesized that psychosocial strengths would be positively associated with subjective well-being, health-related quality of life, and post-traumatic growth. Specifically, all strengths were expected to show positive bivariate associations with these outcomes, and strengths consistently identified in prior RPM research (sense of purpose, psychological endurance, and social support) were hypothesized to predict better functioning in multivariate models controlling for trauma exposure. Associations involving other VIA character strengths were examined on an exploratory basis.

## Methods

### Participants

Participants were 672 English-speaking Canadian adults (69% women, 27% men, 3% non-binary, 1% preferred not to say). Over half (53%) were aged 34 or younger, including 28% aged 18–24. Most identified as White (63%), 17% as Asian, and 20% as other racial or ethnic backgrounds.

### Procedure

The VIA Institute on Character, a nonprofit research organization, administers the VIA-IS, a measure of 24 character strengths freely available on its website. Adults aged 18 and older who completed the survey were invited to participate in a follow-up study. Data were collected over an 8-day period in October–November 2023, yielding 15,495 participants. All provided informed consent, and no incentives were offered. Study procedures were approved by the University of the South Institutional Review Board.

For the present study, the sample was limited to Canadian residents, yielding 847 initial respondents. To ensure data quality, two attention-check items were included. The first, placed midway through the survey, instructed participants to select “Somewhat true about me,” which 82.8% (*n* = 701) answered correctly. The final item asked respondents to choose a response written in capital letters. Participants who answered correctly or had a missing response (with <30% overall missing data) were retained, resulting in a final analytic sample of 672 participants.

### Measures

#### Values in Action questionnaire of character strengths

The VIA Inventory of Strengths–Positive (VIA-IS-P; Peterson and Seligman, 2004) was used to assess 24 character strengths, which are theorized as the behavioral and psychological expressions of broader virtues (Peterson and Seligman, 2004). The scale has been validated across diverse populations (Niemiec, 2013) and demonstrates acceptable internal consistency (mean Cronbach’s α = 0.71; McGrath et al., 2022). The VIA-IS-P is a 96-item self-report measure rated on a 5-point Likert scale ranging from 1 (“Not at all like me”) to 5 (“Very much like me”). Items describe personal attributes such as gratitude, love, and judgment. A complete list of strengths is presented in Tables 1 to 3.

**Table 1.** Blockwise logistic regression of adversities and strengths as predictors of subjective well-being.

	Step 1		Step 2		Step 3	
	Odds ratio		Odds ratio		Odds ratio	
Predictors	Exp(B)	95% CI	Exp(B)	95% CI	Exp(B)	95% CI
*Block 1 – Demographics*						
Age	1.27^***^	1.12–1.43	1.27^***^	1.12–1.43	1.30^*^	1.06–1.58
Household income	1.14^*^	1.00–1.29	1.12	0.99–1.27	1.04	0.84–1.28
Gender – Women vs others	1.34	0.90–1.99	1.38	0.92–2.06	1.03	0.54–1.95
Gender – Non-binary/prefer not to answer vs others	0.76	0.36–1.60	0.81	0.38–1.74	0.97	0.35–2.67
*R*^2^ Demographics only	0.08					
*Block 2 – Adversities*						
Victimization (JVQ)			0.89^*^	0.81–0.98	0.96	0.85–1.08
Institutional betrayal			0.85	0.70–1.02	0.73^*^	0.56–0.95
Δ *R*^2^ when adversities added			0.03			
*R*^2^ Adversities + Demographics			0.11			
*Block 3 – Strengths*						
Meaning making strengths						
Appreciation of Beauty					0.81	0.60–1.10
Gratitude					1.30	0.88–1.92
Honesty					0.98	0.72–1.34
Hope					2.77^***^	1.85–4.17
Perspective					1.35^*^	1.00–1.82
Sense of purpose					1.93^***^	1.33–2.80
Spirituality					0.61^**^	0.43–0.87
Regulatory strengths						
Bravery					0.86	0.64–1.17
Creativity					1.01	0.73–1.41
Humor					1.22	0.93–1.61
Judgment					0.69^*^	0.49–0.97
Mindfulness					1.29	0.98–1.70
Perseverance					0.62^*^	0.43–0.89
Prudence					1.52^*^	1.05–2.19
Psychological endurance					1.51^*^	1.08–2.12
Self-regulation					1.02	0.71–1.46
Zest					1.28	0.91–1.81
Interpersonal strengths						
Fairness					0.84	0.63–1.12
Forgiveness					1.08	0.80–1.46
Kindness					0.85	0.61–1.17
Leadership					1.10	0.80–1.53
Love					1.33	0.98–1.79
Social intelligence					0.72	0.52–1.01
Social support					1.84^***^	1.40–2.42
Teamwork					0.96	0.72–1.28
Environmental strengths						
Eco-connectedness					1.27	0.94–1.70
Strength not assigned to a RPM domain						
Curiosity					0.97	0.68–1.38
Humility					0.85	0.61–1.17
Love of learning					1.00	0.75–1.33
Poly-strengths					1.04	0.94–1.15
Δ *R*^2^ when strengths added					0.43	
Final *R*^2^ full model					0.54	

*Note.* Final full model with all planned variables included. At each block, the percentage of variance explained by the variables in that and any previous block is shown (*R*^2^). For Blocks 2 and 3, the additional variance explained by the new variables is also shown (Δ *R*^2^).

* *p* < 0.05. ^**^p < 0.01. ^***^p < 0.01.

**Table 2.** Blockwise logistic regression of adversities and strengths as predictors of posttraumatic growth.

	Step 1		Step 2		Step 3	
	Odds ratio		Odds ratio		Odds ratio	
Predictors	Exp(B)	95% CI	Exp(B)	95% CI	Exp(B)	95% CI
*Block 1 – Demographics*						
Age	1.13^*^	1.01–1.26	1.12	1.00–1.26	1.00	0.83–1.20
Household income	1.01	0.91–1.12	1.00	0.90–1.11	0.86	0.74–1.01
Gender – Women vs others	1.55^*^	1.05–2.29	1.56^*^	1.05–2.32	1.55	0.84–2.87
Gender – Non-binary/prefer not to answer vs others	0.92	0.48–1.76	0.92	0.47–1.78	0.97	0.35–2.74
*R*^2^ Demographics only	0.03					
*Block 2 – Adversities*						
Victimization (JVQ)			0.97	0.89–1.05	1.05	0.93–1.18
Institutional Betrayal			1.02	0.87–1.20	1.01	0.80–1.28
Δ *R*^2^ when adversities added			0.00			
*R*^2^ Adversities + Demographics			0.03			
*Block 3 – Strengths*						
Meaning making strengths						
Appreciation of beauty					0.90	0.67–1.21
Gratitude					0.86	0.59–1.26
Honesty					0.96	0.72–1.29
Hope					1.60^**^	1.14–2.25
Perspective					1.11	0.84–1.47
Sense of purpose					2.04^***^	1.44–2.89
Spirituality					0.71^*^	0.51–0.99
Regulatory strengths						
Bravery					0.98	0.74–1.30
Creativity					0.87	0.64–1.18
Humor					1.09	0.84–1.40
Judgment					0.72^*^	0.53–0.99
Mindfulness					1.18	0.91–1.52
Perseverance					0.92	0.66–1.28
Prudence					0.89	0.63–1.26
Psychological endurance					1.50^*^	1.09–2.05
Self-regulation					0.94	0.67–1.31
Zest					1.36	0.98–1.88
Interpersonal strengths						
Fairness					0.76^*^	0.58–1.00
Forgiveness					1.31	0.98–1.75
Kindness					0.86	0.63–1.17
Leadership					0.91	0.67–1.23
Love					1.47^**^	1.10–1.95
Social intelligence					0.79	0.58–1.09
Social support					1.59^***^	1.24–2.02
Teamwork					1.05	0.80–1.38
Environmental strengths						
Eco-connectedness					1.06	0.80–1.38
Strength not assigned to a RPM domain						
Curiosity					0.99	0.71–1.37
Humility					1.02	0.79–1.33
Love of learning					1.11	0.85–1.45
Poly-strengths					1.20^***^	1.09–1.32
Δ *R*^2^ Strengths added					0.52	
Final *R*^2^ full model					0.55	

*Note.* Final full model with all planned variables included. At each block, the percentage of variance explained by the variables in that and any previous block is shown (*R*^2^). For Blocks 2 and 3, the additional variance explained by the new variables is also shown (Δ *R*^2^).

* *p* < 0.05. ^**^p < 0.01. ^***^p < 0.01.

**Table 3.** Blockwise logistic regression of adversities and strengths as predictors of health-related quality of life (HRQOL).

	Step 1		Step 2		Step 3	
	Odds ratio		Odds ratio		Odds ratio	
Predictors	Exp(B)	95% CI	Exp(B)	95% CI	Exp(B)	95% CI
*Block 1 – Demographics*						
Age	1.13	0.97–1.30	1.13	0.97–1.33	1.14	0.92–1.40
Household income	1.14	0.98–1.31	1.12	0.96–1.30	1.15	0.96–1.38
Gender – Women vs others	0.62^**^	0.43–0.89	0.61^**^	0.43–0.88	0.65	0.41–1.01
Gender – Non-binary/prefer not to answer vs others	0.70	0.31–1.61	0.79	0.33–1.89	0.91	0.43–1.92
*R*^2^ Demographics only	0.05					
*Block 2 – Adversities*						
Victimization (JVQ)			0.91^*^	0.84–0.99	0.94	0.85–1.04
Institutional Betrayal			0.73^***^	0.61–0.88	0.71^**^	0.57–0.88
Δ *R*^2^ when adversities added			0.05			
*R*^2^ Adversities + Demographics			0.10			
*Block 3 – Strengths*						
Meaning making strengths						
Appreciation of beauty					0.85	0.66–1.08
Gratitude					1.07	0.79–1.45
Honesty					0.78	0.60–1.00
Hope					0.86	0.65–1.15
Perspective					1.23	0.97–1.57
Sense of purpose					1.10	0.83–1.45
Spirituality					0.85	0.64–1.13
Regulatory strengths						
Bravery					1.24	0.96–1.59
Creativity					0.77	0.59–1.01
Humor					1.05	0.85–1.30
Judgment					0.84	0.64–1.09
Mindfulness					1.10	0.88–1.36
Perseverance					1.15	0.87–1.52
Prudence					1.00	0.75–1.34
Psychological endurance					1.07	0.83–1.39
Self-regulation					1.27	0.96–1.68
Zest					1.73^***^	1.30–2.30
Interpersonal strengths						
Fairness					0.90	0.72–1.14
Forgiveness					1.10	0.86–1.40
Kindness					0.83	0.64–1.08
Leadership					0.73*	0.56–0.95
Love					0.97	0.78–1.21
Social intelligence					0.86	0.66–1.12
Social support					0.88	0.72–1.07
Teamwork					1.20	0.95–1.52
Environmental strengths						
Eco-connectedness					0.98	0.77–1.23
Strength not assigned to a RPM domain						
Curiosity					1.17	0.88–1.55
Humility					1.29^*^	1.04–1.60
Love of learning					0.88	0.69–1.11
Poly-strengths					1.07	1.00–1.15
Δ *R*^2^ Strengths added					0.18	
Final *R*^2^ full model					0.28	

*Note.* Final full model with all planned variables included. At each block, the percentage of variance explained by the variables in that and any previous block is shown (*R*^2^). For Blocks 2 and 3, the additional variance explained by the new variables is also shown (Δ *R*^2^).

* *p* < 0.05. ^**^p < 0.01. ^***^p < 0.01.

#### Additional strengths

Five supplementary strengths were added, including three derived from prior RPM research that were not well represented in the VIA framework (Brooks et al., 2024; Hamby et al., 2018, 2020). These included *sense of purpose* (meaning-making domain), *psychological endurance* (regulatory domain), and *social support received* (interpersonal domain). Participants rated each item on a 4-point scale ranging from 1 (“Not true about me”) to 4 (“Mostly true about me”).

The *sense of purpose* scale assesses the extent to which individuals perceive their lives as meaningful (4 items; e.g. “My life has a clear sense of purpose”) and demonstrated high internal consistency (α = 0.84). The *psychological endurance* scale (4 items; e.g. “When hard times come around, I face them head-on”) measures perseverance in the face of adversity and showed acceptable reliability (α = 0.72). The *social support received* scale (4 items; e.g. “Someone was there for me when I was having a hard time”) evaluates perceived assistance and encouragement from others, with excellent reliability in the current sample (α = 0.91).

Two additional measures were drawn from recent RPM research. The *eco-connections* scale (8 items; e.g. “I enjoy being surrounded by nature”) represents the environmental domain of the RPM (Hamby et al., 2022; Manco and Hamby, 2021) and assesses individuals’ sense of attachment and kinship to nature. Internal consistency in the current sample was high (α = 0.85). The *mindfulness* scale (4 items; e.g. “I take time to be quiet in the present moment”) measures awareness of and connection to one’s thoughts and feelings, demonstrating acceptable reliability (α = 0.76).

#### Adversity

Two measures assessed participants’ experiences of adversity. Childhood interpersonal victimization was evaluated using the adapted short form of the *Juvenile Victimization Questionnaire* (8 dichotomous items; α = 0.72), covering peer bullying, physical and online abuse, exposure to interparental violence, and caregiver assault, neglect, and emotional abuse. *Institutional betrayal* was measured with a newly developed 4-item dichotomous scale (α = 0.60) assessing breaches of trust by organizations and professionals, including professional, professional-organizational, corporate, and systemic forms of betrayal.

#### Indicators of functioning

Three indicators of positive functioning were assessed. *Subjective well-being* was measured with four items adapted from prior RPM research (Brooks et al., 2024; e.g. “I have a lot to be proud of”) showing excellent reliability (α = 0.92). *Posttraumatic growth* was evaluated with four items adapted from the Posttraumatic Growth Inventory (Tedeschi and Calhoun, 1996), reflecting positive change following adversity (e.g. “I discovered that I am stronger than I thought I was”; α = 0.85). *Health-related quality of life* was assessed with four items adapted from the CDC’s Healthy Days Measure, which evaluate perceived health status and the extent to which poor health or pain interfered with daily activities. Reliability for this scale was high (α = 0.80).

#### Demographics

Demographic characteristics, including age, gender, and racial and ethnic background was obtained through the standard VIA assessment.

### Data analysis

Missing data were handled using multiple imputation (MI) following recommended guidelines (Graham, 2012). The procedure, conducted in SPSS with the fully conditional specification (MCMC) method and 100 iterations, produced five imputed datasets. Analyses were based on pooled estimates.

Descriptive statistics summarized sample characteristics and victimization frequencies, followed by blockwise logistic regressions informed by prior RPM studies. All scale scores were standardized (z-scores, *M* = 0, *SD* = 1). A poly-strengths index (0–29) was created, assigning one point for each strength exceeding 0.5 SD above the mean. Three blockwise models examined predictors of (1) subjective well-being, (2) post-traumatic growth, and (3) health-related quality of life. Consistent with past studies using the RPM (Brooks et al., 2024; Hamby et al., 2018, 2020), we focused on identifying factors predicting above-average functioning, with subjective well-being, post-traumatic growth and health-related quality of life dichotomized with a score of 1 indicating values above 0.5 SD. Sociodemographics (age, gender, income) were used as covariates and entered first, adversities (victimization, institutional betrayal) second, and poly-strengths with 29 strengths last. Bivariate correlations are provided in Supplemental Material.

## Results

### Frequency of victimization experiences

Most participants (93.2%) reported at least one form of interpersonal victimization, most often peer abuse (exclusion or psychological abuse). Nearly two-thirds (65.2%) had been hit or attacked on purpose, and 42.6% experienced psychological abuse by a parent. About one in three reported childhood physical abuse by an adult or cyberabuse. Exposure to interparental violence affected one in four participants, and one in five experienced caregiver neglect. Half of respondents endorsed at least one form of institutional betrayal, most commonly professional betrayal (38.7%), such as being hurt, threatened, or mistreated by a boss, doctor, or teacher.

### Bivariate analyses

The poly-strengths score was significantly associated with the proportion of participants classified as thriving across all three indicators (subjective well-being: χ^2^ (5, *N* = 672) = 114.87, *p* < 0.001; post-traumatic growth: χ^2^ (5, *N* = 672) 170.60, *p* < 0.001, health-related quality of life: χ^2^ (5, *N* = 672) 27.93, *p* < 0.001). These findings (See Figure 1) indicate a clear gradient effect, whereby increases in the number of strengths are associated with progressively higher proportions of individuals classified as thriving across all outcomes, supporting the cumulative resilience hypothesis central to the RPM.

**Figure 1.**
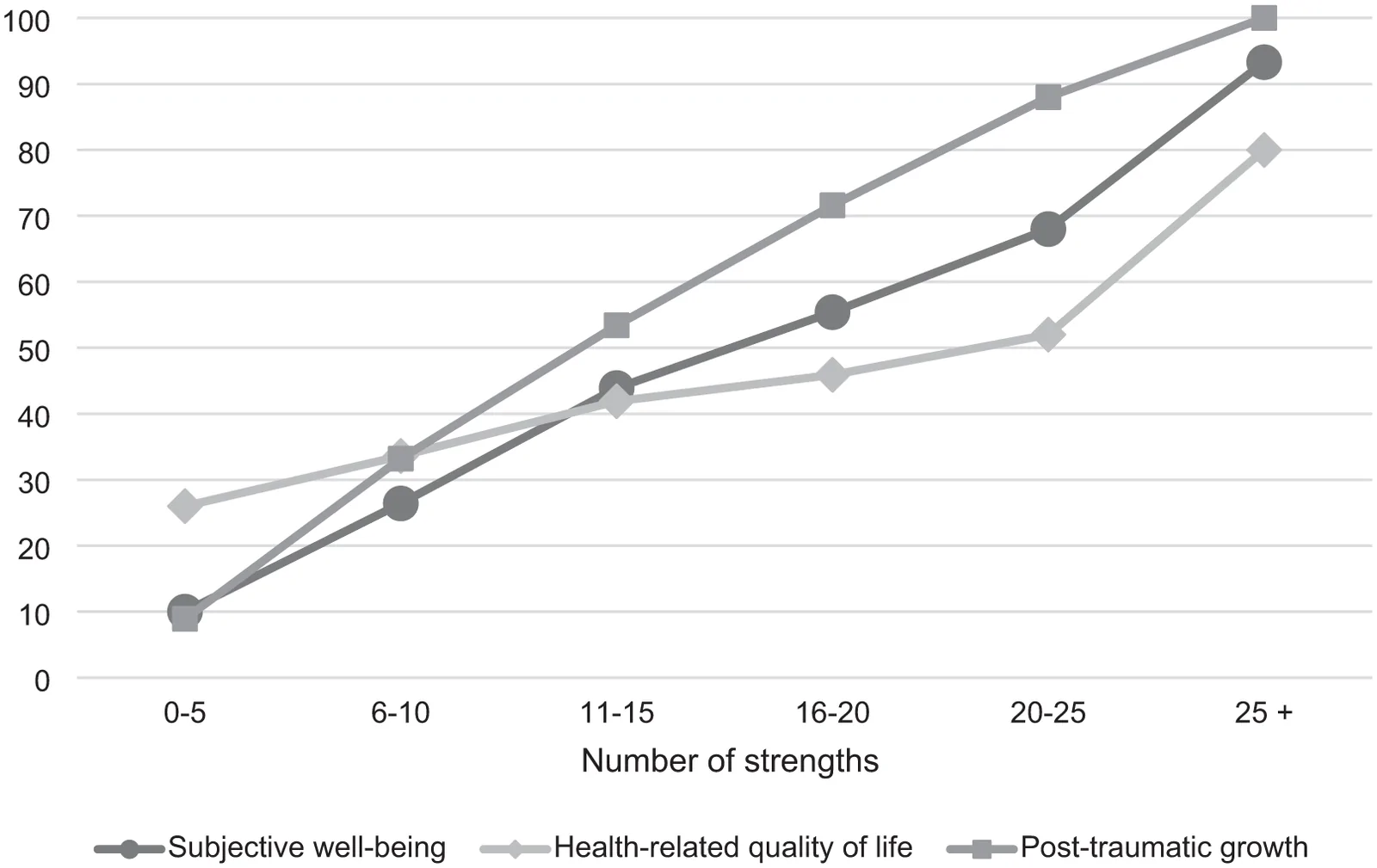
Percentage of participants thriving by number of strengths.

### Blockwise logistic regressions

The first blockwise logistic regression examined predictors of subjective well-being (see Table 1). Sociodemographic variables entered at the first step accounted for 8% of the variance, while adding the adversities experienced in the second block contributed to an additional 3% of the variance in scores of subjective well-being. The inclusion of the different strengths in the last block contributed to an additional 43% of explained variance. In the final model, older participants reported higher well-being, whereas having experienced institutional betrayal was linked to a lower likelihood of thriving. Controlling for sociodemographics and adversities, six strengths made unique contributions. From the meaning-making domain, *hope* (OR = 2.77), *sense of purpose* (OR = 1.93), and *perspective* (OR = 1.35) predicted greater well-being. From the regulatory domain, *psychological endurance* and *prudence* were also significantly associated with greater well-being. Finally, *social support received* (OR = 1.84) from the interpersonal domain was associated with nearly twice the likelihood of thriving. Unexpectedly, *judgment*, *perseverance*, and *spirituality* were linked to lower odds of well-being.

A second blockwise regression examined predictors of post-traumatic growth (Table 2). Sociodemographic variables and adversities together explained 3% of the variance, while strengths accounted for an additional 52%. After controlling for prior blocks, post-traumatic growth was significantly predicted by the poly-strengths score and five individual strengths. From the meaning-making domain, *sense of purpose* (OR = 2.04) and *hope* (OR = 1.60) showed the strongest effects. *Psychological endurance* (regulatory domain) and two interpersonal strengths, *social support received* and *love*, were also significant predictors in the expected direction. Unexpectedly, higher scores in *judgment*, *spirituality*, and *fairness* were associated with a lower likelihood of thriving in terms of post-traumatic growth.

The third logistic regression examined predictors of health-related quality of life (Table 3). Sociodemographic variables explained 5% of the variance, adversities added 5% more, with institutional betrayal predicting poorer outcomes. Adding strengths accounted for a further 18% of explained variance. In the final model, *zest* and *humility* increased the odds of thriving, whereas *leadership* was linked to a lower likelihood of reporting good health-related quality of life.

## Discussion

The present study explored the relationship between character strengths, adversity, and global functioning indicators. A total of 29 strengths derived from the VIA survey, along with the RPM, were used to assess subjective well-being, post-traumatic growth, and health-related quality of life in a sample of English-speaking Canadians. The findings underscore the role of personal strengths in predicting well-being following adversity. Our study offers a different perspective on resilience research, providing a valuable insight to the predictive variables associated with thriving in the face of lifecourse trauma.

The results provide significant insight into the frequency of victimization experiences, and the factors that predict subjective well-being, health-related quality of life, and post-traumatic growth. They reinforce the impact of interpersonal adversity (especially trauma in childhood) on well-being, while also highlighting the substantial role of personal strengths in fostering resilience, providing a deeper understanding of how individual navigate adversity and thrive despite challenges. Specifically, psychological strengths, namely *sense of purpose*, *hope* and *psychological endurance*, emerged as the most significant predictors of resilience across multiple indicators, including subjective well-being and post-traumatic growth.

A key finding in this study was the central role of meaning-making strengths, such as *hope* and *sense of purpose*, in predicting subjective well-being. *Hope* is defined as the ability to derive purpose, significance, or meaning from life experiences. It enables the individual to believe in their ability to work toward and achieve future goals, thereby motivating actions that enhance the likelihood of achieving desired outcomes (Peterson and Seligman, 2004). Hope has strongly been associated with greater life satisfaction, improved psychological adjustment, and the use of positive coping strategies (Dorsett et al., 2017). One possible explanation is the mediating role of hope between cognitive reappraisal and adaptive emotion regulation (Leite et al., 2019). Cognitive reappraisal is a self-regulatory cognitive process in which individuals reinterpret emotionally charged situations to modulate their emotional responses (Gross and Jazaieri, 2014). By facilitating constructive emotion regulation, hope acts as an essential mechanism for psychological resilience and subjective well-being. A sense of purpose can be understood as having both meaning in one’s daily activities and a broader perception that life itself is meaningful. The literature has linked a strong sense of purpose to various benefits, including better physical health (e.g. lower risk of mortality), healthier behaviors (e.g. increased physical activity and reduced sleep problems), improved psychosocial outcomes (e.g. lower negative affect), reduced risk of depression, and decreased loneliness over a 4-year follow-up in a representative sample of U.S. adults (Kim et al., 2022). Individuals with a higher sense of purpose have also been found to manage daily stressors more effectively (Hill et al., 2018), exhibit lower impulsivity (Burrow and Spreng, 2016), demonstrate greater self-efficacy (Rush et al., 2021), and recover more quickly from negative emotional stimuli (Schaefer et al., 2013).

Two additional strengths were found to predict both subjective well-being and post-traumatic growth: *psychological endurance* and *social support received. Psychological endurance* is defined here as the ability to serve as a reliable source of strength for one’s family or community in times of need. It involves remaining true to oneself, even in the face of adversity or exposure to interpersonal trauma (Hamby et al., 2018). In this sense, endurance has a social component, reflecting the extent to which others can depend on an individual during difficult times. In contrast, this distinguishes it from perseverance, which is more goal-oriented and typically refers to persistence in completing specific tasks (Biggs et al., 2024). Endurance has been associated with positive psychological outcomes (Brooks et al., 2024; Gonzalez-Mendez et al., 2021; Hamby et al., 2018). Furthermore, a synthesis of 60 meta-analyses found a strong association between social support and psychological adjustment across a wide range of outcomes and age groups (Zell and Stockus, 2025). This association remained robust regardless of the source of social support (e.g. parents, friends, teachers, colleagues, supervisors). Therefore, perceived social support thus serves as a protective factor against psychological distress, regardless of the specific social circle from which it originates.

Some strengths were uniquely associated with a single indicator of functioning, further emphasizing the diverse ways in which protective factors contribute to positive adaptation. *Prudence*, defined as the ability to plan actions and decisions to achieved future goals, was linked to increasing likelihood of thriving in terms of subjective well-being. Similarly, *perspective*, characterized by the wisdom and self-awareness that enables a balanced approach to decision making, was also linked to subjective well-being. *Love*, encompassing a complex blend of cognitive, behavioral, and emotional orientation toward others, was associated with a higher probability of thriving in terms of post-traumatic growth (Peterson and Seligman, 2004).

Regarding health-related quality of life, the results indicated that *zest* and *humility* were identified as predictors of thriving. *Zest*, qualified by vitality and eagerness to engage in life (Peterson and Seligman, 2004), has been linked to various aspects of enhanced well-being. People with higher zest tend to adopt more positive health habits (e.g. healthy eating, regular exercise) which contribute to improved health-related quality of life specifically (Weziak-Bialowolska et al., 2023). *Humility*, defined as the belief that something greater than oneself exists, is associated with acceptance of one’s strength and limitations (Peterson and Seligman, 2004). This acceptance has been identified as an effective coping strategy for humble individuals while dealing with their symptoms, making them more likely to rate their health-related quality of life positively (Wilski et al., 2019). These variables were related to a wide variety of health outcomes in a previous study (Weziak-Bialowolska et al., 2023). However, the limited explanatory power of these variables suggests that numerous additional factors likely shape individuals’ perceptions of health-related quality of life. Social determinants of health, including social, economic, and environmental variables play a crucial part in influencing health outcomes and overall quality of life (Chung et al., 2022). This demonstrates that health-related quality of life is not solely determined by individual traits, further explaining the small variance accounted for by the study variables.

Unexpectedly, some strengths were associated with lower resilience, underscoring the complexity of these constructs and their contextual impact on adaptation. Among these, *spirituality* and *judgment* showed significant negative associations with both well-being and post-traumatic growth, such that higher levels of these strengths were associated with lower odds of thriving, contrary to their positive bivariate correlations. This pattern suggests that when multiple strengths are considered simultaneously, spirituality and judgment may no longer function as independent protective factors; instead, strengths, such as sense of purpose, hope, and social support may exert more robust influences.

The finding for judgment is consistent with recent work indicating that justice-related character strengths are less frequently associated with well-being than meaning-making or relational strengths (Niemiec, 2023), suggesting a more indirect or context-dependent role in resilience processes. More broadly, shifts in the direction of associations observed in multivariate models may reflect suppression or competitive effects among correlated strengths (Gaylord-Harden et al., 2010). In the case of spirituality, this may capture complex facets that can both support and constrain adaptation, such as engagement in spiritual communities that provide meaning but may also introduce stress when aspects of one’s identity are not affirmed. Consistent with this interpretation, maladaptive forms of religious coping (e.g. construing adversity as divine punishment) have been linked to poorer adjustment (Puchalska-Wasyl and Małaj, 2024). Taken together, these findings align with evidence that overreliance on any single strength may carry unintended costs (Weziak-Bialowolska et al., 2023) and highlight the importance of examining strengths within the broader configuration of an individual’s resilience portfolio.

To situate the present findings within the emerging Canadian literature on strengths and resilience, we compare our results with prior Canadian studies examining related constructs in younger and more context-specific samples. Our findings can be contrasted with those of Fang et al. (2025) and Moisan et al. (2019), which similarly examined the association between psychological strengths and subjective well-being in Canadian populations. These studies, however, were geographically and developmentally more limited, focusing on adolescents in Quebec (Moisan et al., 2019) and in university students in Montreal (Fang et al., 2025), whereas our sample was more geographically diverse. Several strengths showed convergence across studies. Our measure of *perspective* characterized by wisdom and balanced decision-making, parallels the *reflective tendency* reported by Fang et al. (2025). While *perspective* was positively associated with well-being in our study, *reflective tendency* was not in Fang et al.′s work and was instead linked to adverse experiences. Contextual and developmental differences may help explain this divergence, as younger or university-based samples may engage in both adaptive and maladaptive meaning-making processes following adversity. In contrast, participants in our broader adult sample may reflect more constructively, contributing to better well-being. Despite these differences, findings converge across studies regarding the protective role of social support. Finally, *optimism* identified by Moisan et al. (2019) aligns closely with *hope* in the present study, with both strengths emerging as significant predictors of well-being across Canadian contexts.

Building on the empirical findings of the present study, these results have broader implications for how strengths-based resilience is conceptualized and applied in the Canadian context. The current study extends an understanding of strengths and resilience in a Canadian context. Projects like the Canadian index of well-being and national progress indicators (Muhajarine et al., 2012; Wilson and Tyedmers, 2013) that go beyond simple economic definitions of flourishing are gaining attention. Many of these projects work to measure a diversity of outcomes that may indicate thriving. Research like the current study helps to identify groups of strengths that may help contribute to those broader national indices and can be important modifiable factors to promote well-being and national indicators of progress.

From a theoretical standpoint, the present findings extend both the RPM and the VIA framework. For the RPM, results provide empirical support for a portfolio-based conceptualization of resilience, demonstrating that psychosocial strengths account for substantially more variance in thriving outcomes than adversity exposure once trauma has occurred. For the VIA framework, findings underscore that character strengths are context-dependent and not uniformly protective in trauma-exposed populations. Certain strengths may overlap with or be subsumed by broader meaning-making and relational resources, while others may function differently depending on cumulative strength profiles. Together, these results suggest that resilience is best conceptualized as an interaction between multiple strengths rather than the sum of isolated traits.

### Limitations

This study has several limitations. Not all forms of interpersonal victimization were assessed, and the focus on childhood experiences may not capture the full range of violence adults have faced. Strengths fostering adaptation may also vary across gender, cultural, and social groups, highlighting the need for more inclusive, context-sensitive approaches. The cross-sectional design limits causal inference, and reliance on self-report may introduce recall or social desirability bias, particularly for sensitive topics. Future research should consider sexual and gender diversity, as distinct forms of victimization and coping strengths may emerge. Building on the present analytic approach, person-oriented and network analyses could also offer deeper insights by identifying distinct constellations and interconnections of strengths that support adaptation, beyond what aggregated data reveal. Future research could also explore other ways of operationalizing thriving and assessing the constructs examined here.

### Implications for practice

The present findings offer several implications for clinical practice and prevention. Incorporating strength-based assessments, rather than focusing solely on symptoms, may allow for a more comprehensive understanding of an individual’s resilience despite adversity. By identifying strengths such as psychological endurance or social support, practitioners can develop targeted interventions that build on these assets rather than solely addressing deficits. Strengths-based assessment aligns closely with evidence-based trauma treatments by complementing symptom-focused approaches with identification of existing adaptive resources. Interventions such as trauma-focused cognitive behavioral therapy and narrative interventions explicitly target meaning-making, emotion regulation, and social connection, domains reflected in the strengths identified in the present study. Integrating strengths assessment into these approaches may enhance engagement, personalize intervention targets, and support resilience-oriented recovery trajectories.

At the individual level, interventions including mindfulness, psychoeducation, and narrative interventions have been shown to strengthen meaning-making processes such as sense of purpose (Manco and Hamby, 2021). Narrative interventions that emphasize values, turning points, and meaningful life events may be particularly well suited to fostering purpose following adversity. Mindfulness-based approaches may also reduce victimization risk (Hamby et al., 2025). In the Canadian context, mindfulness and compassion-based interventions, including emerging digital and immersive formats, are being developed and evaluated with promising results (Spinelli et al., 2023).

From a prevention perspective, strengths can be intentionally nurtured across development. Opportunities for goal setting, engagement in meaningful activities, and the formation of supportive relationships with adults and peers may foster hope, purpose, and social connection, resources shown to promote resilience later in life. School-based programs such as the FRIENDS for Life program exemplify how social support and coping skills can be cultivated early to reduce risk and promote adaptive trajectories (Rose et al., 2009).

At broader systemic levels, resilience-oriented practice can be reinforced by ensuring equitable access to strength-building opportunities within communities, such as affordable extracurricular activities. At the population level, incorporating strength-based indicators into health and well-being surveillance may improve understanding of resilience across regions and inform policy. Overall, shifting the focus from deficits to strengths offers a complementary pathway for supporting thriving following adversity.

## Supplemental Material

sj-docx-1-hpq-10.1177_13591053261433238 – Supplemental material for Identifying factors associated with thriving after interpersonal trauma in a Canadian sample of adults Supplemental material, sj-docx-1-hpq-10.1177_13591053261433238 for Identifying factors associated with thriving after interpersonal trauma in a Canadian sample of adults by Martine Hébert, Victoria Banyard, Xiafei Wang, Ophélie Dassylva, Stéphanie El Asmar and Sherry Hamby in Journal of Health Psychology

## Data Availability

The dataset used during the current study is available from the corresponding author on reasonable request.*
