# Supplementary material for: Identifying factors associated with thriving after interpersonal trauma in a Canadian sample of adults
Source: J Health Psychol. 2026 Mar 30;31(13):5814–33. doi: 10.1177/13591053261433238 (PMC13683900; doi:10.1177/13591053261433238)
Supplement: Supplementary file 1 [file sj-docx-1-hpq-10.1177_13591053261433238.docx]

Supplemental material

*Correlations Among Strengths and Indicators of Functioning*

|  | Subjective well-being | Post-traumatic growth | Health-related quality of life |
| --- | --- | --- | --- |
| *Meaning making strengths* |  |  |  |
| Appreciation of Beauty | .195*** | .247*** | .025 |
| Gratitude | .553*** | .469*** | .268*** |
| Honesty | .288*** | .263*** | .087* |
| Hope | .643*** | .563*** | .305*** |
| Perspective | .326*** | .284*** | .123*** |
| Sense of Purpose | .603*** | .585*** | .219*** |
| Spirituality | .330*** | .381*** | .077* |
| *Regulatory strengths* |  |  |  |
| Bravery | .234*** | .283*** | .004 |
| Creativity | .194*** | .238*** | -.007 |
| Humor | .190*** | .212*** | .060 |
| Judgment | .138*** | .080* | .071 |
| Mindfulness | .356*** | .367*** | .190*** |
| Perseverance | .275*** | .268*** | .302*** |
| Prudence | .183*** | .106** | .170*** |
| Psychological Endurance | .391*** | .386*** | .202*** |
| Self-Regulation | .345*** | .287*** | .279*** |
| Zest | .536*** | .518*** | .345*** |
| *Interpersonal strengths* |  |  |  |
| Fairness | .193*** | .148*** | .078* |
| Forgiveness | .316*** | .293*** | .176*** |
| Kindness | .176*** | .254*** | .010 |
| Leadership | .334*** | .303*** | .106** |
| Love | .376*** | .408*** | .154*** |
| Social Intelligence | .313*** | .334*** | .126*** |
| Social Support | .413*** | .424*** | .082** |
| Teamwork | .279*** | .334*** | .238*** |
| *Environmental strengths* |  |  |  |
| Eco-connectedness | .224*** | .224*** | .052 |
| *Strength not assigned to a RPM domain* |  |  |  |
| Curiosity | .293*** | .312*** | .116** |
| Humility | .071 | .097* | .049 |
| Love of Learning | .253*** | .252*** | .136*** |
|  |  |  |  |
|  |  |  |  |

*Note.* ^*^ *p* < .05; ^**^ *p* < .01; ^***^ *p* < .01.
